# Effect of Lemongrass Essential Oil Vapors on Microbial Dynamics and *Listeria monocytogenes* Survival on Rocket and Melon Stored under Different Packaging Conditions and Temperatures

**DOI:** 10.3390/microorganisms3030535

**Published:** 2015-09-09

**Authors:** Agni Hadjilouka, Melissanthi Polychronopoulou, Spiros Paramithiotis, Periklis Tzamalis, Eleftherios H. Drosinos

**Affiliations:** Laboratory of Food Quality Control and Hygiene, Department of Food Science and Human Nutrition, Agricultural University of Athens, Iera Odos 75, GR-118 55 Athens, Greece; E-Mails: agni_xatz@aua.gr (A.H.); melisshome@hotmail.com (M.P.); sdp@aua.gr (S.P.); pertzam@aua.gr (P.T.)

**Keywords:** *Listeria monocytogenes*, lemongrass, essential oils, fresh-cut salads, modified atmospheres

## Abstract

The aim of the present study was to examine the effect of lemongrass essential oil vapors on the dynamics of surface microbiota and *L*. *monocytogenes* growth on rocket and melon under different packaging conditions and storage temperature. For that purpose, rocket and melon were placed on Expanded Polystyrene (EPS) trays, sprayed with *L*. *monocytogenes* to a population of 4.5–5.0 log CFU·g^−1^, packaged using microperforated Oriented Polypropylene (OPP) film in either air or Microperforated Active Modified Atmosphere (MAMA) (initial atmosphere 5% O_2_, 10% CO_2_) including a Whatman paper containing the essential oil, without contact with the product, and stored at 0, 5, 10, and 15 °C. Application of lemongrass exhibited a bactericidal effect on enterococci and a fungistatic effect on yeast-mould populations but only during air storage of rocket. The former took place at all temperatures and the latter only at 10 and 15 °C. No effect on shelf life of both products was recorded. However, an important effect on the sensorial properties was observed; during the first 4–5 days of storage both products were organoleptically unacceptable. Regarding MAMA packaging, it affected only *Pseudomonas* spp. population resulting in a reduction of 1–2 log CFU·g^−1^ in both products.

## 1. Introduction

*Listeria monocytogenes* has been implicated as the causative agent in several foodborne outbreaks after consumption of ready-to-eat fruits and vegetables. Contamination of fresh produce is facilitated by the ubiquitous nature of the pathogen as it is widely distributed in soil surface, decaying vegetation, soil sewage, animal feces, river and canal waters, fertilizers, plants, and animals and it is known to survive in plant material for 10–12 years [1] and in soil for up to 295 days [2]. Therefore fruits and vegetables can become easily contaminated with the pathogen while growing in fields or greenhouses, during harvesting or post-harvest handling [3].

Protection of fresh produce against pathogenic microorganisms needs to be carried out through treatments that do not preclude the food from bearing the term “fresh”, which suggests or implies that the food is unprocessed, it is in a raw state and has not been frozen or subjected to any form of thermal processing or any other form of preservation [4]. Therefore, improvement of mild preservation techniques and use of natural antimicrobial compounds have been widely studied over the last decades [5]. Among the natural antimicrobials that are used in food industry are the essential oils (EO’s), volatile oily liquids obtained from different plant parts and fruits. The antimicrobial properties of the essential oils have been known for centuries. They are widely used in food industry as food flavors and numerous studies have described their antimicrobial effects as well as their great importance in several other fields, such as pharmacology and pharmaceutics [6,7,8].

The inherent aroma and antimicrobial activity of EO’s are usually related to the concentration and chemical structure of their components and also to the interactions among the components that are affecting their bioactive properties [9]. Their main advantage is that they can be used in any foods and that the U.S Food and Drug Administration have classified these substances as Generally Recognized As Safe (GRAS status) or as approved food additives [10,11]. Furthermore they are widely used in culinary practices, a fact that makes them easily accepted by consumers. However, EOs’ antimicrobial efficacy is usually achieved in concentrations that inflict changes in the natural taste and odor of the food [12]. In order to reduce this impact, the use of vapors instead of the direct addition has been proposed [13]. In vapor contact assays, EO’s are not in direct contact with the food and yet microbial inhibition against foodborne pathogens and spoilage microorganisms is achieved. The inhibition in these cases is achieved at relatively lower concentrations compared to liquid phase application and therefore the effect on sensory characteristics is reduced [9,14].

Lemongrass (*Cymbopogon citratus*) is a tall perennial grass, widely cultivated in warm tropical and subtropical regions [15]. It contains 1% to 2% essential oil on a dry basis and its chemical composition varies as a function of genetic diversity, habitat, and agronomic treatment of the culture [16]. The volatile oil obtained from the fresh leaves of this grass is widely used in the perfume and cosmetic industries. It is mostly composed of monoterpene compounds; citral is a major component, which is a natural mixture of two isomeric acyclic monoterpene aldehydes: geranial and neral. Apart from citral, the oil also consists of myrcene, geraniol, and geranyl acetate [17]. It has been speculated that EOs without phenolic groups, such as lemongrass oil, cause membrane disruption due to their lipophilic compounds [18]. Lemongrass oil does not only damage the membrane structure through monoterpene diffusion but also facilitates solubility in cell membranes when applied in gaseous form [19,20]. Furthermore, lemongrass essential oil has antidepressant, antioxidant, antiseptic, astringent, nervine, sedative as well as bactericidal, fungicidal, and generally antimicrobial activity against a diverse range of microorganisms including moulds and yeasts, Gram-positive and negative bacteria [15,21,22,23,24]. However, only a limited number of studies have been performed regarding the application of essential oil in fresh produce [25,26,27] with very promising results.

Modified atmosphere packaging (MAP) is widely used for fresh-cut produce preservation. The factors that determine the gas composition of a MAP include the respiration rate and the respiring area of the product, the storage temperature, O_2_ and CO_2_ permeabilities of the packaging materials *etc*. [28,29,30]. Two major problems are associated with MAP, the time required for the build-up of the desired atmosphere in the case of passive or equilibrium MAP, and the possibility of oxygen depletion in the case of active MAP. Both may be confronted with the approach termed Microperforated Active Modified Atmosphere (MAMA) packaging in which the active MAP is combined with the use of a microperforated film [31].

However, there is a lack in the literature concerning the effect of this type of packaging on the surface microbiota as well as foodborne pathogen dynamics, especially in the presence of essential oils that exert antimicrobial action. Therefore, the aim of the present study was to examine the effect of lemongrass oil vapors on surface microbiota and *L. monocytogenes* growth on rocket salad and melon packaged in air and MAMA conditions and stored at 0, 5, 10, and 15 °C.

## 2. Materials, Methods and Data Treatments

### 2.1. Bacterial Strains and Culture Conditions

*L. monocytogenes* strain LQC 15257; belonging to serotype 4b, previously isolated from a strawberry sample was used throughout this study. The strain was *in vitro* sensitive to the vapors of lemongrass essential oil. Long-term storage took place at −20 °C in nutrient broth supplemented with 50% glycerol. Before experimental use, the strain was grown twice in Brain Heart Infusion broth (Biolife, Milan, Italy) at 37 °C for 24 h. Inoculum preparation took place as follows: overnight culture (9 log CFU·mL^−1^) was centrifuged (12,000× *g*; 10 min; 4 °C), washed twice with sterile saline, resuspended in the same diluent and used to inoculate rocket and melon samples at 4.5–5.0 log CFU·g^–1^ through serial dilutions.

### 2.2. Essential Oil

The essential oil (EO) of lemongrass (*Cymbopogon citratus*) was purchased by Kokkinakis Essans E.P.E. (Athens, Greece).

### 2.3. In situ Impact of Lemongrass Oil Vapors on Microbiota Development

Rocket (*Eruca sativa*) and melon (*Cucumis melo* cultivar Honeydew) cubes (approx. dimensions 3 × 3 × 4 cm) were packaged as follows: 30 g of rocket or 50 g of melon were placed on Expanded Polystyrene (EPS) trays, sprayed or not with 0.5 mL of appropriately diluted pathogen population to obtain the desired final population (4.5–5.0 log CFU·g^−1^). Holes were punched in the EPS tray in order to allow the essential oil volatiles to reach as much surface of the product as possible. Then 100 μL of the essential oil was applied on a Whatman paper that was then placed outside of the tray, *i.e*. without any contact with the product. Packaging took place using microperforated, Oriented Polypropylene (OPP) film (O_2_ permeability: 8000 cm^3^/m^2^·24 h·atm) in either air or modified atmosphere (5% O_2_, 10% CO_2_) and stored at 0, 5, 10, and 15 °C.

Sampling was performed every 24 h. The atmosphere (%O_2_ & CO_2_) within the packaging was measured using a CheckMate 9900 O_2_/CO_2_, (PBI Dansensor A/S, Ringsted, Denmark). Total aerobic mesophilic, yeasts-molds, *Enterobacteriaceae*, enterococci, *Pseudomonas* spp., lactic acid bacteria and *L. monocytogenes* counts were determined according to Paramithiotis *et al*. [32]. Finally, a panel of non-specialists was used to evaluate the appearance, texture, aroma and overall quality of the product. The freshness of the product was rated according to a 1–5 rating scale were a score of 3 was considered to be the limit of marketability [33].

### 2.4. Statistical Analysis

The experiment was performed in triplicate. One-way analysis of variance (ANOVA) was used to statistically assess the effect of MAMA packaging and lemongrass essential oil’s addition to shelf life and microbial population dynamics.

## 3. Results and Discussion

In Table 1 the microbiota population dynamics as well as the limit of marketability of packaged rocket salad during incubation at 0, 5, 10, and 15 °C, with and without application of lemongrass essential oil and with or without inoculation with *L. monocytogenes* are presented.

Rocket salad reached the limit of marketability on average after 2, 3, 10, and 10.5 days of storage in air at 15, 10, 5, and 0 °C, respectively. The initial atmosphere within the packaging consisted of 20.8% O_2_ and 0.0% CO_2_. After the first day of storage, equilibration took place and the atmosphere within the packaging consisted of 18%–20% O_2_ and 0.00%–2.65% CO_2_ and was stable throughout storage. During storage in MAMA, rocket reached the limit of marketability on average after 2.5, 4, 9 and 12.5 days at 15, 10, 5, and 0 °C, respectively. The initial atmosphere within the packaging consisted of 5% O_2_ and 10% CO_2_. After the first day of storage, equilibration took place and the atmosphere within the packaging consisted of 17.65%–20.80% O_2_ and 0.15%–3.30% CO_2_ and was stable throughout storage. Application of lemongrass essential oil seemed to have no effect on the shelf life of the product stored in both atmosphere conditions.

The initial microbiota of the rocket salad was dominated by yeasts-molds that ranged between 5.07 and 6.36 log CFU·g^−1^. Enterococci population ranged between below enumeration limit to 3.20 log CFU·g^−1^, while *Enterobacteriaceae* and *Pseudomonas* spp. were enumerated at 3.20–4.40 and 3.90–5.00 log CFU·g^−1^, respectively. Lactic acid bacteria were below the enumeration limit and absence of *L. monocytogenes* was verified. At the end of storage period *Pseudomonas* spp. prevailed the microecosystem of rocket salads stored in air reaching populations of 7.97, 8.40, 8.28, and 8.98 log CFU·g^−1^ at 0, 5, 10, and 15 °C, respectively. On the contrary, *Pseudomonas* spp. co-dominated with yeasts during storage at MAMA mostly due to decrease in the *Pseudomonas* spp. population. More accurately, in all temperatures MAMA packaging resulted in a *ca*. 1 log CFU·g^−1^ decrease of the *Pseudomonas* spp. population.

Application of lemongrass in air packaged rocket salads seemed to have a bactericidal effect against enterococci. Indeed, when lemongrass essential oil was applied enterococci populations were below the enumeration limit; on the contrary, they reached a population range of 2–5 log CFU·g^−1^ without application of lemongrass essential oil. The same effect was observed regarding yeasts-molds growth at 10 and 15 °C; without lemongrass essential oil application the population reached 7 log CFU·g^−1^ but when the essential oil was applied the population would not exceed 5.5 log CFU·g^−1^. However, this was not the case regarding the application of the essential oil in MAMA packaged rocket salads; enterococci as well as yeast-mold populations were not affected. No significant effect on the growth of *Enterobacteriaceae* and *Pseudomonas* spp. in both air and MAMA packaged rocket salads was also observed when lemongrass essential oil was applied.

Finally, *L. monocytogenes* population remained stable at all temperatures throughout storage time.

Melon reached the limit of marketability on average after 4, 7, 9, and 13 days of storage in air at 15, 10, 5, and 0 °C, respectively. The initial atmosphere within the packaging consisted of 20.8% O_2_ and 0.0% CO_2._ After the first day of storage, equilibration took place and the atmosphere within the packaging consisted of 18.40%–19.90% O_2_ and 1.15%–2.60% CO_2_, during storage at 15 °C and 20.05%–20.80% O_2_ and 0.00%–0.65% CO_2_, during storage at 0, 5 and 10 °C that were stable throughout storage. During storage in MAMA, melon reached the limit of marketability on average after 4, 7, 9, and 15 days at 15, 10, 5, and 0 °C, respectively. The initial atmosphere within the packaging consisted of 5% O_2_ and 10% CO_2._ After the first day of storage, equilibration took place and the atmosphere within the packaging consisted of 18.45%–19.80% O_2_ and 1.10%–2.75% CO_2_, during storage at 15 °C and 19.10%–20.80% O_2_ and 0.00%–0.85% CO_2_, during storage at 0, 5 and 10 °C that were stable throughout storage. As in the case of rocket salad, application of lemongrass essential oil seemed to have no effect on shelf life of the product.

Melon initial microbiota was dominated by yeasts-molds and *Pseudomonas* spp. that ranged between 3.54–3.95 and 3.00–3.61 log CFU·g^−1^, respectively. *Enterobacteriaceae*, enterococci and lactic acid bacteria were below the enumeration limit and absence of *L. monocytogenes* was verified. At the end of storage period, *Pseudomonas* spp. prevailed with the microecosystem of melon stored in air reaching populations of 5.65, 7.50, 7.66, and 7.52 log CFU·g^−1^ at 0, 5, 10 and 15 °C, respectively. On the contrary, *Pseudomonas* spp. co-dominated with yeasts during storage at MAMA mostly due to a decrease in the *Pseudomonas* spp. population. More accurately, in all temperatures MAMA packaging resulted in a *ca*. 1 log CFU·g^−1^ decrease of the *Pseudomonas* spp. population.

*L. monocytogenes* remained stable between 3.6 and 4.3 log CFU·g^−1^ during storage at 0 °C. However, at 5, 10, and 15 °C growth was observed and population of the pathogen ranged between 5.2 and 6.9 logCFU·g^−1^. In general, application of lemongrass essential oil seemed to have no effect in the microbial categories studied (Table 2).

**Table 1:** Limit of marketability (days) and microbial population dynamics (log CFU g^−1^) during incubation of rocket at 0, 5, 10, and 15°C packaged in air or MAMA conditions, with or without lemongrass essential oil, with or without inoculation with *L. monocytogenes*.

Parameter	Air		Air & EO ^1^		Air & Lm ^2^		Air & EO & Lm		MAMA		MAMA & EO		MAMA & Lm		MAMA & EO & Lm	
	Initial ^3^	Final ^4^	Initial	Final	Initial	Final	Initial	Final	Initial	Final	Initial	Final	Initial	Final	Initial	Final
0 °C																
LoM ^5^	10		11		10		11		12		12		13		13	
Yeasts-molds	5.70 (0.44) ^a^	6.50 (0.41) ^a^	6.24 (0.37) ^a^	6.69 (0.08) ^a^	6.32 (0.11) ^a^	6.70 (0.47) ^a^	5.93 (0.02) ^a^	6.01 (0.24) ^a^	5.50 (0.30) ^a^	6.05 (0.43) ^a^	5.45 (0.63) ^a^	6.02 (0.45) ^a^	5.69 (0.36) ^a^	6.58 (0,52) ^b^	5.60 (0.36) ^a^	6.30 (0.22) ^b^
Enterococci	3.15 (0.15) ^a^	3.31 (0.28) ^a^	<2	<2	2.20 (0.10) ^a^	2.68 (0.40) ^a^	2.95 (0.54) ^a^	<2 ^b^	2.54 (0.15) ^a^	3.54 (0.38) ^b^	2.32 (0.10) ^a^	2.42 (0.12) ^a^	2.77 (0.25) ^a^	3.20 (0.21) ^b^	2.77 (0.24) ^a^	3.58 (0.41) ^b^
*Enterobacteriaceae*	3.59 (0.11)^a^	4.33 (0.30) ^b^	3.80 (0.10) ^a^	4.42 (0.38) ^a^	3.30 (0.34) ^a^	4.40 (0.20) ^b^	3.39 (0.28) ^a^	4.40 (0.25) ^b^	3.20 (0.20) ^a^	4.57 (0.32) ^b^	4.40 (0.17) ^a^	5.10 (0.25) ^b^	4.30 (0.50) ^a^	4.50 (0.30) ^a^	3.80 (0.20) ^a^	4.30 (0.27) ^b^
*Pseudomonas* spp.	4.50 (0.30) ^a^	7.90 (0.23) ^b^	4.56 (0.32) ^a^	7.97 (0.29) ^b^	4.25 (0.21) ^a^	7.40 (0.30) ^b^	4.36 (0.66) ^a^	7.32 (0.24) ^b^	4.50 (0.20) ^a^	6.80 (0.39) ^b^	4.02 (0.53) ^a^	6.40 (0.40) ^b^	4.50 (0.24) ^a^	6.60 (0.25) ^b^	5.00 (0.63) ^a^	6.80 (0.45) ^b^
LAB ^6^	<2	<2	<2	<2	<2	<2	<2	<2	<2	<2	<2	<2	<2	<2	<2	<2
*L. monocytogenes*	absence	absence	absence	absence	3.96 (0.33) ^a^	3.68 (0.50) ^a^	3.55 (0.49) ^a^	3.20 (0.24) ^a^	absence	absence	absence	absence	4.50 (0.39) ^a^	4.21 (0.44) ^a^	4.20 (0.11) ^a^	3.80 (0.30) ^a^
TAMC ^7^	6.46 (0.20) ^a^	8.50 (0.27) ^b^	6.30 (0.04) ^a^	8.51 (0.60) ^b^	6.69 (0.35) ^a^	8.20 (0.25) ^b^	5.98 (0.23) ^a^	8.20 (0.23) ^b^	5.52 (0.53) ^a^	7.80 (0.20) ^b^	5.66 (0.36) ^a^	7.75 (0.32) ^b^	6.30 (0.36) ^a^	7.42 (0.42) ^b^	6.39 (0.30) ^a^	7.23 (0.23) ^b^
5 °C																
LoM	9		10		11		10		8		9		9		10	
Yeasts-molds	5.80 (0.04) ^a^	6.50 (0.26) ^b^	5.07 (0.07) ^a^	6.66 (0.33) ^b^	5.25 (0.31) ^a^	6.10 (0.18) ^a^	5.93 (0.12) ^a^	6.39 (0.54) ^a^	5.86 (0.32) ^a^	6.32 (0.54) ^a^	5.58 (0.36) ^a^	6.00 (0.55) ^a^	5.69 (0.52) ^a^	6.86 (0.22) ^b^	5.69 (0.33) ^a^	6.50 (0.65) ^a^
Enterococci	3.15 (0.15) ^a^	2.30 (0.10) ^b^	2.47 (0.12) ^a^	<2 ^b^	2.20 (0.12) ^a^	2.60 (0.30) ^a^	2.92 (0.32) ^a^	<2 ^b^	2.46 (0.15) ^a^	3.68 (0.58) ^b^	2.20 (0.15) ^a^	2.40 (0.20) ^a^	2.77 (0.24) ^a^	2.40 (0.22) ^a^	2.77 (0.36) ^a^	2.30 (0.22) ^a^
*Enterobacteriaceae*	3.59 (0.11) ^a^	5.40 (0.68) ^b^	3.80 (0.10) ^a^	4.85 (0.15) ^a^	3.30 (0.34) ^a^	4.32 (0.14) ^b^	3.39 (0.58) ^a^	4.80 (0.54) ^a^	3.20 (0.58) ^a^	4.85 (0.54) ^b^	4.20 (0.36) ^a^	5.24 (0.44) ^b^	4.30 (0.25) ^a^	4.80 (0.42) ^a^	3.30 (0.32) ^a^	4.81 (0.32) ^b^
*Pseudomonas* spp.	4.50 (0.25) ^a^	8.40 (0.26) ^b^	4.70 (0.41) ^a^	8.20 (0.35) ^b^	4.25 (0.41) ^a^	8.35 (0.41) ^b^	4.50 (0.65) ^a^	8.25 (0.45) ^b^	4.40 (0.36) ^a^	6.80 (0.50) ^b^	3.90 (0.32) ^a^	7.15 (0.25) ^b^	4.85 (0.20) ^a^	7.30 (0.32) ^b^	4.22 (0.20) ^a^	7.05 (0.33) ^b^
LAB	<2	<2	<2	<2	<2	<2	<2	<2	<2	<2	<2	<2	<2	<2	<2	<2
*L. monocytogenes*	absence	absence	absence	absence	4.20 (0.30) ^a^	4.37 (0.20) ^a^	4.55 (0.49) ^a^	3.87 (0.42) ^a^	absence	absence	absence	absence	4.50 (0.21) ^a^	4.12 (0.33) ^a^	4.50 (0.32) ^a^	4.00 (0.23) ^a^
TAMC	6.40 (0.20) ^a^	8.73 (0.24) ^b^	5.38 (0.04) ^a^	8.27 (0.11) ^b^	6.69 (0.35) ^a^	8.50 (0.21) ^b^	6.81 (0.71) ^a^	8.74 (0.36) ^b^	5.85 (0.26) ^a^	8.14 (0.36) ^b^	6.10 (0.53) ^a^	7.92 (0.36) ^b^	6.39 (0.65) ^a^	8.12 (0.58) ^b^	6.39 (0.36) ^a^	7.20 (0.25) ^b^
10 °C																
LoM	3		3		3		3		4		4		4		4	
Yeasts-molds	5.45 (0.08) ^a^	7.54 (0.24) ^b^	5.50 (0.17) ^a^	4.47 (0.48) ^b^	5.80 (0.08) ^a^	7.13 (0.12) ^b^	5.50 (0.15) ^a^	4.60 (0.30) ^b^	6.34 (0.52) ^a^	7.35 (0.39) ^b^	5.89 (0.65) ^a^	7.46 (0.45) ^b^	5.80 (0.25) ^a^	7.20 (0.52) ^b^	5.90 (0.56) ^a^	7.40 (0.54) ^b^
Enterococci	3.20 (0.17) ^a^	4.46 (0.06) ^b^	3.15 (0.15) ^a^	<2 ^b^	2.47 (0.08) ^a^	3.34 (0.14) ^b^	2.41 (0.10) ^a^	<2 ^b^	2.89 (0.11) ^a^	3.52 (0.29) ^b^	2.82 (0.15) ^a^	3.32 (0.26) ^b^	2.53 (0.32) ^a^	2.80 (0.30) ^a^	2.50 (0.32) ^a^	2.52 (0.38) ^a^
*Enterobacteriaceae*	3.60 (0.11) ^a^	4.79 (0.08) ^b^	3.39 (0.08) ^a^	4.60 (0.10) ^b^	3.80 (0.10) ^a^	4.44 (0.39) ^b^	3.41 (0.10) ^a^	4.51 (0.03) ^a^	3.56 (0.24) ^a^	4.16 (0.07) ^b^	3.31 (0.49) ^a^	4.47 (0.40) ^b^	3.80 (0.52) ^a^	4.20 (0.32) ^a^	3.60 (0.36) ^a^	4.00 (0.32) ^a^
*Pseudomonas* spp.	4.25 (0.06) ^a^	8.28 (0.10) ^b^	4.50 (0.47) ^a^	8.25 (0.29) ^b^	4.25 (0.58) ^a^	8.05 (0.27) ^b^	4.35 (0.47) ^a^	8.17 (0.60) ^b^	4.56 (0.12) ^a^	7.32 (0.26) ^b^	4.86 (0.41) ^a^	7.36 (0.35) ^b^	4.23 (0.25) ^a^	7.13 (0.52) ^b^	4.00 (0.25) ^a^	7.53 (0.36) ^b^
LAB	<2	<2	<2	<2	<2	<2	<2	<2	<2	<2	<2	<2	<2	<2	<2	<2
*L. monocytogenes*	absence	absence	absence	absence	4.19 (0.11) ^a^	4.20 (0.23) ^a^	4.42 (0.05) ^a^	4.31 (0.20) ^a^	absence	absence	absence	absence	4.20 (0.23) ^a^	4.63 (0.28) ^a^	4.20 (0.42) ^a^	4.62 (0.52) ^a^
TAMC	5.80 (0.75) ^a^	8.30 (0.15) ^b^	6.17 (0.32) ^a^	8.27 (0.21) ^b^	5.89 (0.76) ^a^	8.65 (0.06) ^b^	5.89 (0.36) ^a^	8.51 (0.07) ^b^	6.68 (0.42) ^a^	8.13 (0.39) ^b^	5.85 (0.55) ^a^	7.65 (0.46) ^b^	5.86 (0.36) ^a^	7.62 (0.52) ^b^	6.20 (0.51) ^a^	7.60 (0.48) ^b^
15 °C																
LoM	2		2		2		2		2		3		2		3	
Yeasts-molds	5.44 (0.08) ^a^	7.82 (0.27) ^b^	5.46 (0.15) ^a^	5.30 (0.20) ^a^	5.68 (0.08) ^a^	7.30 (0.30) ^b^	5.46 (0.15) ^a^	5.18 (0.27) ^a^	5.36 (0.53) ^a^	7.20 (0.37) ^b^	5.71 (0.64) ^a^	7.47 (0.37) ^b^	5.90 (0.19) ^a^	7.30 (0.08) ^b^	5.52 (0.16) ^a^	7.39 (0.14) ^b^
Enterococci	3.15 (0.15) ^a^	5.78 (0.18) ^b^	3.15 (0.15) ^a^	<2 ^b^	2.47 (0.14) ^a^	2.30 (0.20) ^a^	2.41 (0.10) ^a^	<2 ^b^	2.67 (0.11) ^a^	2.52 (0.21) ^a^	2.57 (0.19) ^a^	3.77 (0.14) ^b^	2.52 (0.23) ^a^	2.75 (0.68) ^a^	2.52 (0.23) ^a^	3.90 (0.41) ^b^
*Enterobacteriaceae*	3.59 (0.11) ^a^	4.91 (0.07) ^b^	3.54 (0.10) ^a^	4.86 (0.07) ^b^	3.80 (0.10) ^a^	4.55 (0.13) ^b^	3.35 (0.10) ^a^	4.80 (0.07) ^b^	3.87 (0.43) ^a^	4.68 (0.46) ^a^	3.84 (0.44) ^a^	4.80 (0.32) ^b^	4.10 (0.23) ^a^	4.89 (0.24) ^b^	3.74 (0.59) ^a^	4.38 (0.20) ^a^
*Pseudomonas* spp.	4.50 (0.58) ^a^	8.33 (0.18) ^b^	4.23 (0.47) ^a^	8.98 (0.05) ^b^	4.25 (0.32) ^a^	8.05 (0.58) ^b^	4.32 (0.47) ^a^	8.85 (0.09) ^b^	4.24 (0.14) ^a^	7.37 (0.19) ^b^	4.21 (0.34) ^a^	7.48 (0.42) ^b^	4.24 (0.14) ^a^	7.57 (0.07) ^b^	4.55 (0.25) ^a^	7.98 (0.45) ^b^
LAB	<2	<2	<2	<2	<2	<2	<2	<2	<2	<2	<2	<2	<2	<2	<2	<2
*L. monocytogenes*	absence	absence	absence	absence	4.19 (0.11) ^a^	4.55 (0.49) ^a^	4.39 (0.03) ^a^	4.25 (0.12) ^a^	absence	absence	absence	absence	4.62 (0.13) ^a^	4.45 (0.14) ^a^	4.66 (0.10) ^a^	4.77 (0.35) ^a^
TAMC	5.80 (0.75) ^a^	8.41 (0.10) ^b^	5.70 (0.32) ^a^	8.87 (0.04) ^b^	5.80 (0.76) ^a^	8.30 (0.15) ^b^	6.20 (0.32) ^a^	8.69 (0.17) ^b^	6.33 (0.46) ^a^	8.08 (0.27) ^b^	6.67 (0.66) ^a^	8.63 (0.63) ^b^	6.57 (0.21) ^a^	8.12 (0.37) ^b^	6.49 (0.38) ^a^	8.26 (0.36) ^b^

^1^ EO: Essential oil; ^2^ Lm: *Listeria monocytogenes*; ^3^ initial: initial counts designate the microbial counts enumerated upon packaging; ^4^ final: microbial counts enumerated at the day designated as limit of marketability; ^5^ LoM: limit of marketability; ^6^ LAB: Lactic acid bacteria; ^7^ TAMC: Total aerobic mesophilic count. The standard deviation is given in parentheses. Within a row, different letters (^a^ or ^b^) between initial and final population of a microbial population denote significant differences (α < 0.05).

**Table 2:** Limit of marketability (days) and microbial population dynamics (log CFU·g^−1^) during incubation of melon at 0, 5, 10, and 15 °C packaged in air or MAMA conditions, with or without lemongrass essential oil, with or without inoculation with *L. monocytogenes*.

Parameter	Air		Air & EO ^1^		Air & Lm ^2^		Air & EO & Lm		MAMA		MAMA & EO		MAMA & Lm		MAMA & EO & Lm	
	Initial ^3^	Final ^4^	Initial	Final	Initial	Final	Initial	Final	Initial	Final	Initial	Final	Initial	Final	Initial	Final
0 °C																
LoM ^5^	13		13		13		13		15		15		15		15	
Yeasts-molds	3.90 (0.25) ^a^	3.60 (0.25) ^a^	3.90 (0.25) ^a^	3.80 (0.23) ^a^	3.95 (0.35) ^a^	3.66 (0.24) ^a^	3.80 (0.15) ^a^	4.05 (0.42) ^a^	3.90 (0.25) ^a^	4.26 (0.22) ^a^	3.60 (0.45) ^a^	4.10 (0.16) ^a^	3.92 (0.56) ^a^	4.06 (0.51) ^a^	3.74 (0.12) ^a^	4.20 (0.23) ^b^
Enterococci	<2	<2	<2	<2	<2	<2	<2	<2	<2	<2	<2	<2	<2	<2	<2	<2
*Enterobacteriaceae*	<1	<1	<1	<1	<1	<1	<1	<1	<1	<1	<1	<1	<1	<1	<1	<1
*Pseudomonas spp.*	3.20 (0.32) ^a^	5.20 (0.36) ^b^	3.17 (0.12) ^a^	5.20 (0.35) ^b^	3.50 (0.14) ^a^	5.60 (0.40) ^b^	3.15 (0.39) ^a^	5.65 (0.54) ^b^	3.20 (0.32) ^a^	4.60 (0.23) ^b^	3.33 (0.12) ^a^	4.66 (0.21) ^b^	3.50 (0.30) ^a^	5.00 (0.24) ^b^	3.29 (0.15) ^a^	4.45 (0.28) ^b^
LAB ^6^	<2	<2	<2	<2	<2	<2	<2	<2	<2	<2	<2	<2	<2	<2	<2	<2
*L. monocytogenes*	absence	absence	absence	absence	3.77 (0.20) ^a^	4.30 (0.22) ^b^	3.10 (0.20) ^a^	4.20 (0.23) ^b^	absence	absence	absence	absence	3.87 (0.21) ^a^	3.69 (0.23) ^a^	3.65 (0.20) ^a^	3.88 (0.42) ^a^
TAMC ^7^	4.77 (0.30) ^a^	5.80 (0.36) ^b^	4.77 (0.30) ^a^	5.60 (0.32) ^b^	4.77 (0.30) ^a^	5.80 (0.36) ^b^	5.00 (0.30) ^a^	5.80 (0.21) ^b^	4.60 (0.23) ^a^	5.30 (0.34) ^b^	4.40 (0.30) ^a^	5.30 (0.32) ^b^	4.82 (0.34) ^a^	5.23 (0.43) ^a^	4.34 (0.32) ^a^	5.15 (0.22) ^b^
5 °C																
LoM	9		9		9		9		9		9		9		9	
Yeasts-molds	3.72 (0.05) ^a^	5.20 (0.35) ^b^	3.80 (0.20) ^a^	5.50 (0.32) ^b^	3.92 (0.12) ^a^	5.40 (0.36) ^b^	3.68 (0.25) ^a^	5.60 (0.35) ^b^	3.81 (0.15) ^a^	5.20 (0.32) ^b^	3.63 (0.45) ^a^	5.50 (0.42) ^b^	3.95 (0.05) ^a^	5.40 (0.32) ^b^	3.80 (0.07) ^a^	5.20 (0.36) ^b^
Enterococci	<2	3.30 (0.21)	<2	3.02 (0.21)	<2	3.33 (0.21)	<2	2.80 (0.32)	<2	3.40 (0.21)	<2	3.62 (0.23)	<2	3.35 (0.21)	<2	3.62 (0.23)
*Enterobacteriaceae*	<1	4.30 (0.41)	<1	4.70 (0.42)	<1	4.22 (0.21)	<1	4.20 (0.32)	<1	4.30 (0.32)	<1	4.51 (0.32)	<1	4.20 (0.32)	<1	4.50 (0.33)
*Pseudomonas* spp.	3.02 (0.17) ^a^	7.45 (0.23) ^b^	3.17 (0.12) ^a^	7.40 (0.36) ^b^	3.20 (0.20) ^a^	7.50 (0.24) ^b^	3.10 (0.08) ^a^	7.50 (0.32) ^b^	3.41 (0.13) ^a^	5.60 (0.35) ^b^	3.32 (0.41) ^a^	5.54 (0.22) ^b^	3.16 (0.27) ^a^	5.60 (0.24) ^b^	3.10 (0.42) ^a^	5.60 (0.44) ^b^
LAB	<2	4.50 (0.22)	<2	4.60 (0.33)	<2	4.40 (0.35)	<2	4.38 (0.35)	<2	4.25 (0.33)	<2	4.37 (0.33)	<2	4.20 (0.36)	<2	4.52 (0.33)
*L. monocytogenes*	absence	absence	absence	absence	3.77 (0.05) ^a^	5.30 (0.52) ^b^	3.77 (0.05) ^a^	5.40 (0.36) ^b^	absence	absence	absence	absence	3.77 (0.05) ^a^	5.60 (0.42) ^b^	3.77 (0.05) ^a^	5.20 (0.32) ^b^
TAMC	4.77 (0.30) ^a^	7.60 (0.36) ^b^	4.77 (0.30) ^a^	7.58 (0.47) ^b^	4.77 (0.30) ^a^	7.80 (0.35) ^b^	4.77 (0.30) ^a^	7.80 (0.24) ^b^	4.77 (0.30) ^a^	7.68 (0.35) ^b^	4.77 (0.30) ^a^	8.15 (0.32) ^b^	4.77 (0.30) ^a^	7.90 (0.35) ^b^	4.77 (0.30) ^a^	7.96 (0.35) ^b^
10 °C																
LoM	7		7		7		7		7		7		7		7	
Yeasts-molds	3.92 (0.15) ^a^	5.42 (0.35) ^b^	3.58 (0.17) ^a^	5.42 (0.36) ^b^	3.72 (0.21) ^a^	5.24 (0.36) ^b^	3.73 (0.35) ^a^	5.62 (0.32) ^b^	3.60 (0.40) ^a^	6.24 (0.45) ^b^	3.73 (0.35) ^a^	6.57 (0.33) ^b^	3.68 (0.20) ^a^	6.85 (0.42) ^b^	3.90 (0.05) ^a^	6.74 (0.33) ^b^
Enterococci	<2	4.30 (0.25)	<2	3.40 (0.30)	<2	3.80 (0.33)	<2	4.06 (0.35)	<2	4.00 (0.30)	<2	3.40 (0.62)	<2	3.80 (0.30)	<2	4.30 (0.35)
*Enterobacteriaceae*	<1	4.63 (0.52)	<1	4.50 (0.32)	<1	4.20 (0.36)	<1	5.10 (0.32)	<1	4.60 (0.33)	<1	4.60 (0.33)	<1	4.66 (0.36)	<1	4.63 (0.52)
*Pseudomonas* spp.	3.31 (0.30) ^a^	7.39 (0.35) ^b^	3.12 (0.10) ^a^	7.66 (0.51) ^b^	3.53 (0.27) ^a^	7.63 (0.32) ^b^	3.42 (0.12) ^a^	7.56 (0.33)^b^	3.61 (0.23) ^a^	6.08 (0.34) ^b^	3.25 (0.17) ^a^	6.22 (0.35) ^b^	3.18 (0.22) ^a^	6.38 (0.42) ^b^	3.40 (0.32) ^a^	6.33 (0.42) ^b^
LAB	<2	4.20 (0.32)	<	4.32 (0.41)	<2	4.30 (0.32)	<2	4.30 (0.33)	<2	4.20 (0.32)	<2	4.63 (0.32)	<2	4.20 (0.32)	<2	4.30 (0.32)
*L. monocytogenes*	absence	absence	absence	absence	3.77 (0.32) ^a^	6.90 (0.32) ^b^	3.77 (0.32) ^a^	6.50 (0.24) ^b^	absence	absence	absence	absence	3.77 (0.32) ^a^	6.54 (0.32) ^b^	3.77 (0.32) ^a^	6.23 (0.32) ^b^
TAMC	4.77 (0.30) ^a^	7.50 (0.62) ^b^	4.77 (0.30) ^a^	7.80 (0.35) ^b^	4.77 (0.30) ^a^	8.12 (0.45) ^b^	4.77 (0.30) ^a^	8.12 (0.26) ^b^	4.77 (0.30)^a^	7.24 (0.35) ^b^	4.77 (0.30) ^a^	7.12 (0.35) ^b^	4.77 (0.30) ^a^	6.86 (0.52) ^b^	4.77 (0.30) ^a^	6.90 (0.23) ^b^
15 °C																
LoM	4		4		4		4		4		4		4		4	
Yeasts-molds	3.83 (0.19) ^a^	6.33 (0.35) ^b^	3.68 (0.17) ^a^	6.68 (0.35) ^b^	3.80 (0.14) ^a^	6.36 (0.50) ^b^	3.92 (0.14) ^a^	6.20 (0.51) ^b^	3.76 (0.23) ^a^	6.50 (0.32) ^b^	3.75 (0.17) ^a^	6.56 (0.35) ^b^	3.80 (0.36) ^a^	6.50 (0.41) ^b^	3.54 (0.22) ^a^	6.30 (0.32) ^b^
Enterococci	<2	5.10 (0.52)	<2	4.88 (0.41)	<2	5.22 (0.67)	<2	4.85 (0.47)	<2	4.58 (0.62)	<2	4.89 (0.24)	<2	5.04 (0.26)	<2	5.33 (0.50)
*Enterobacteriaceae*	<1	5.25 (0.36)	<1	5.50 (0.25)	<1	5.68 (0.41)	<1	5.53 (0.25)	<1	5.68 (0.41)	<1	5.32 (0.24)	<1	5.40 (0.54)	<1	5.30 (0.54)
*Pseudomonas* spp.	3.20 (0.32) ^a^	7.32 (0.42) ^b^	3.12 (0.36) ^a^	7.52 (0.26) ^b^	3.00 (0.23) ^a^	7.52 (0.35) ^b^	3.25 (0.14) ^a^	6.90 (0.54) ^b^	3.12 (0.12) ^a^	6.32 (0.36) ^b^	3.00 (0.23) ^a^	6.20 (0.32) ^b^	3.20 (0.20) ^a^	6.20 (0.24) ^b^	3.21 (0.12) ^a^	6.00 (0.35) ^b^
LAB	<2	4.23 (0.42)	<2	4.12 (0.21)	<2	3.69 (0.14)	<2	4.10 (0.24)	<2	4.30 (0.36)	<2	3.98 (0.41)	<2	3.90 (0.24)	<2	3.90 (0.14)
*L. monocytogenes*	absence	absence	absence	absence	3.77 (0.32) ^a^	6.12 (0.21) ^b^	3.84 (0.24) ^a^	6.47 (0.32) ^b^	absence	absence	absence	absence	3.77 (0.21) ^a^	6.69 (0.24) ^b^	3.77 (0.21) ^a^	6.20 (0.25) ^b^
TAMC	4.27 (0.30) ^a^	7.20 (0.30) ^b^	4.18 (0.25) ^a^	6.90 (0.52) ^b^	4.25 (0.30) ^a^	7.75 (0.07) ^b^	4.57 (0.12) ^a^	7.39 (0.52) ^b^	4.25 (0.25) ^a^	7.12 (0.35) ^b^	4.35 (0.25) ^a^	7.20 (0.24) ^b^	4.27 (0.32) ^a^	7.10 (0.50) ^b^	4.40 (0.25) ^a^	7.12 (0.22) ^b^

^1^ EO: Essential oil; ^2^ Lm: *Listeria monocytogenes*; ^3^ initial: initial counts designate the microbial counts enumerated upon packaging; ^4^ final: microbial counts enumerated at the day designated as limit of marketability; ^5^ LoM: limit of marketability; ^6^ LAB: Lactic acid bacteria; ^7^ TAMC: Total aerobic mesophilic count. The standard deviation is given in parentheses. Within a row, different letters (^a^ or ^b^) between initial and final population of a microbial population denote significant differences (α < 0.05).

In all cases, application of lemongrass essential oil had also an important effect on the sensorial properties, which was more pronounced during the first 4–5 days of storage and faded as storage proceeded, most probably due to evaporation. Indeed, during the first 4–5 days the product was not acceptable by all panelists due to the intense odor, but after that time the residual odor was less intense and the product was more acceptable.

In recent years there has been a remarkable interest in extracts and essential oils from aromatic plants with antimicrobial activities against foodborne pathogens and toxin producing microorganisms [34]. This interest was strongly supported by the fact that essential oils are natural products that can be used as natural additives in many foods due to their antibacterial, antifungal, antioxidant, and anti-carcinogenic properties [35]. The antimicrobial properties of an extended variety of plant oils or extracts have been extensively studied over the last decades. Numerous studies currently exist on the comparison of the effectiveness of different substances against foodborne pathogens and variability in the conclusions drawn has been observed, even for the same essential oils. This can be attributed to a series of factors with the varying composition according to local climatic and environmental conditions [36,37] and the varying origin [38,39] being the most important. The latter should always be kept in mind when assessing the antimicrobial activity of essential oils. In this study, the effect of lemongrass oil vapors on microbiota dynamics and *L. monocytogenes* growth in rocket salads and melons during storage in air and MAMA conditions and at 0, 5, 10, and 15 °C was examined.

Only a limited amount of studies currently exist regarding the effect of MAMA packaging on the shelf life, the dynamics of the surface microbiota as well as the survival of foodborne pathogens in fresh cut fruits and vegetables and, to the best of our knowledge, the first time that such study is performed in *E. sativa* and Honeydew melon. Lokke *et al*. [40] studied the effect of high (48,400 cm^3^/m^2^ 24 h·atm) and low (1900 cm^3^/m^2^ 24 h·atm) oxygen transmission rate (OTR) air packaging on the freshness and sensory quality of wild rocket. It was concluded that high OTR films should be selected when storage temperature cannot be controlled as it allows sufficient aerobic respiration and prevents loss of leaf integrity and texture. In the present study, medium OTR was selected fulfilling this requirement, as indicated by the measurements of the atmosphere in the packaging. Both packaging conditions, *i.e*., air and initial displacement with modified atmosphere (5% O_2_, 10% CO_2_) resulted in comparable shelf life of the products under study, most probably due to the equilibration reached already from the first day of storage at all temperatures assessed. However, this displacement significantly affected the dominating surface microbiota as well as the effect of the lemongrass essential oil. In the first case, *Pseudomonas* spp. dominated the surface microbiota when air was used in both rocket and melon. On the contrary, the initial displacement resulted in co-domination with yeasts-molds. This co-domination resulted from the 1–2 log CFU·g^−1^ reduction of the *Pseudomonas* spp. population, compared to the respective in air. This reduction can be attributed to the initial displacement of air with increased CO_2_ concentration since *Pseudomonas* spp. are known to be among the most sensitive microorganisms to CO_2_ [41].

Several studies have reported antimicrobial activity of lemongrass against fungi and bacteria such as *Acinetobacter baumanii*, *Aeromonas veronii*, *Aspergillus niger, Bacillus cereus, B. subtilis, Botrytis cinerea, Colletotrichum coccodes, Corynebacterium equii, Cladosporium herbarum, Enterococcus faecalis, Enterobacter aerogenes, Escherichia coli, Fusarium verticillioides, Klebsiella pneumoniae, Proteus vulgaris, Rhizopus stolonifer, Salmonella* Typhimurium, *Serratia marcesens*, and *Staphylococcus aureus*, either by agar diffusion method or/and broth dilution [15,21,22,23,24,42,43,44,45]. Contrary to these results, Adegoke and Odesola [46] reported that *Fusarium verticillioides* growth was not affected when lemongrass oil was added in culture medium. Moreover, the essential oil of *C. citratus* was reported as more effective than synthetic fungicides like Agrosan GN, Dithane M-43, and copper oxychloride [42,46].

Although there have been many reports of the antifungal and antibacterial effects of essential oils *per se*, there are much fewer reports on essential oil vapors and particularly on lemongrass vapors. *In vitro* studies on tomato fruits have indicated complete growth inhibition of *Bacillus cinerea* and *Alternaria arborescens* by lemongrass vapors. Moreover *Geotrichum candidum* was reported as more sensitive to citral and citral-containing oil vapors than to thyme and oregano oils. On the other hand, no inhibition was reported for *Rhizopus stolonifer* [43].

The main concern about essential oils’ use is their effect on the sensorial properties of the treated products. Despite the fact that MICs (minimum inhibitory concentrations) of the most active EOs are very low, they can still alter the organoleptic properties of the treated foods [11]. In addition, *in vitro* studies do not necessarily indicate how effective an EO treatment will be when applied in food systems [47]. Indeed, higher concentrations are usually required in order to obtain a similar antimicrobial effect when used in food systems [6]. This is due to interactions between phenolic compounds and the food matrix [48] and should be considered for commercial applications. Therefore, it is necessary to test the EOs on food samples in order to evaluate their actual impact on sensorial characteristics of the specific products. In the present study, lemongrass vapors had an important effect on the sensorial properties of the products under study, especially during the first 4–5 days of their storage due to the intense odor of the oil. Arrebola *et al*. [25] managed to reduce the intense odor of lemongrass oil and retain the quality of peaches during storage, demonstrating the potential of using *Bacillus amyloliquefaciens* in combination with lemongrass oil in a pad delivery system within a biodegradable MAP. By combining *B. amyloliquefaciens* with lemongrass oil, a reduction of the amount of the latter in the delivery system was managed, also reducing the unpleasant odor and taste resulting from the oil.

Lemongrass oil has also been found effective against *Listeria innocua*, *E. coli* and *Salmonella* Enteritidis in apple, pear and melon juices at 35 °C [27]. In the latter study, complete inhibition of the microbial growth was achieved by 2 μL·mL^−1^ in apple and pear juices and by 5 μL·mL^−1^ in melon and tryptone soy broth, highlighting the effect of the food matrix. Azarakhsh *et al*. [26] studied the effects of lemongrass incorporated into alginate-based edible coating for fresh-cut pineapple and reported a significant reduction of the total plate as well as yeast and mold counts of the coated samples during low-temperature storage and a concomitant increase of the shelf life of the product.

## 4. Conclusions

In the present study, application of lemongrass in air packaged rocket salads seemed to have a significant bactericidal effect on growth of yeast-molds at 10–15 °C and in enterococci populations at all temperatures. Moreover, application of lemongrass essential oil seemed to have no effect in the microbial categories studied in rocket salad packaged in MAMA conditions and in melon packaged in both air and MAMA conditions. *L. monocytogenes* population remained stable during storage in air packaged salads at 0, 5, 10, and 15 °C and MAMA packaged rocket salads stored at 0 °C. However, at 5, 10, and 15 °C growth of the pathogen was observed. This may be primarily attributed to the microperforated packaging that allowed the escape of essential oil vapors but was necessary in order to allow sufficient plant tissue respiration. In conclusion, the antimicrobial activity of lemongrass essential oil seemed to be affected by the food matrix and the storage conditions. Moreover, no effect on shelf life of rocket or melon was observed.
